# In Silico Design of a Chimeric Protein Containing Antigenic Fragments of Helicobacter Pylori; A Bioinformatic Approach

**DOI:** 10.2174/1874285801711010301

**Published:** 2017-11-16

**Authors:** Amir Ghasemi

**Affiliations:** Department of Microbiology and Immunology, Faculty of Medicine, Kashan University of Medical Sciences, Kashan, Iran

## REVISED VERSION NOVEMBER 2017

The following list provides a description of the changes made to the publication since the original version of the article entitled “**In Silico Design of a Chimeric Protein Containing Antigenic Fragments of Helicobacter pylori; A Bioinformatic Approach**” was published online in May 2016.

## PAGE 97:

### In the Abstract, the Following Text Appears:

“In order to obtain such benefit in H. pylori vaccine study, a chimeric gene containing four fragments of FliD sequence (1-600 bp), UreB (327-334 bp),VacA (744-805 bp) and CagL(51-100 bp) which have a high density of B- and T-cell epitopes was designed.”

### This Should be:

“In order to obtain such benefit in H. pylori vaccine study, a chimeric gene containing four fragments of FliD sequence (1-600 bp), UreB (327-385 bp),VacA (744-805 bp) and CagL(51-100 bp) which have a high density of B- and T-cell epitopes was designed.”

## PAGE 98:

### In the paragraph 4, the following text appears:

“Urease B has been widely investigated as a potential antigen for the development of prophylactic and therapeutic vaccines against H. pylori infection [32, 33]. UreB(327-334) is considered as a good B cell epitope and has been found to be protective in mice [34, 35]”

### This Should be:

“Urease B has been widely investigated as a potential antigen for the development of prophylactic and therapeutic vaccines against *H. pylori* infection [32, 33]. UreB (327-385) is considered as a good B cell epitope and has been found to be protective in mice [34, 35].”

## PAGE 100:

### In the Results, the Following Text Appears:

“The nominated sequences for designing of chimeric construct were FliD (1-600), UreB (327-334), VacA (744-805) and CagL (51-100).”

### This Should be:

“The nominated sequences for designing of chimeric construct were FliD (1-600), UreB (327-385), VacA (744-805) and CagL (51-100).”

## PAGE 102:

### In the Paragraph 3, the Following Text Appears:

“The secondary structure of the chimeric protein was predicted by several online programs, and the best result was achieved by GOR-IV as shown in Fig. (4). Results indicated total residues of 785 which were made up 140 strands (17.83%), 299 helices (38.09%) and 346 random coils (44.08%). No predicted signal peptide was identified in the initial region of the protein sequence.”

### This Should be:

“The secondary structure of the chimeric protein was predicted by several online programs, and the best result was achieved by GOR-IV as shown in Fig. (**4**). Results indicated total residues of 790 which were made up 140 strands (17.72%), 299 helices (37.85%) and 351 random coils (44.43%). No predicted signal peptide was identified in the initial region of the protein sequence.”

## PAGE 107:

### In the Discussion, the Following Text Appears:

Based on our finding, a chimeric protein including immunodominant epitopes from different antigenic proteins such as FliD (1-600), UreB (327-334), VacA (744-805) and CagL (51-100) would likely induce strong comprehensive protective immunity.

### This Should be:

Based on our finding, a chimeric protein including immunodominant epitopes from different antigenic proteins such as FliD (1-600), UreB (327-385), VacA (744-805) and CagL (51-100) would likely induce strong comprehensive protective immunity.

## PAGE 108:

### In the Conclusion, the Following Text Appears:

“Our data showed that the possibility of successful production of a large chimeric protein composing of four domains FliD (1-600), UreB(327-334), VacA (744-805) and CagL (51-100) in the prokaryotic host.”

### This Should be:

“Our data showed that the possibility of successful production of a large chimeric protein composing of four domains FliD (1-600), UreB(327-385), VacA (744-805) and CagL (51-100) in the prokaryotic host.”

